# Plant and Microalgae Derived Peptides Are Advantageously Employed as Bioactive Compounds in Cosmetics

**DOI:** 10.3389/fpls.2019.00756

**Published:** 2019-06-12

**Authors:** Fabio Apone, Ani Barbulova, Maria Gabriella Colucci

**Affiliations:** ^1^Arterra Bioscience srl, Naples, Italy; ^2^Vitalab srl, Naples, Italy

**Keywords:** bioactive, peptide, plant, microalgae, cosmetics

## Abstract

Bioactive peptides (BP) are specific protein fragments that are physiologically important for most living organisms. It is proven that in humans they are involved in a wide range of therapeutic activities as antihypertensive, antioxidant, anti-tumoral, anti-proliferative, hypocholesterolemic, and anti-inflammatory. In plants, BP are involved in the defense response, as well as in the cellular signaling and the development regulation. Most of the peptides used as ingredients in health-promoting foods, dietary supplements, pharmaceutical, and cosmeceutical preparations are obtained by chemical synthesis or by partial digestion of animal proteins. This makes them not fully accepted by the consumers because of the risks associated with solvent contamination or the use of animal derived substances. On the other hand, plant and microalgae derived peptides are known to be selective, effective, safe, and well tolerated once consumed, thus they have got a great potential for use in functional foods, drugs, and cosmetic products. In fact, the interest in the plant and microalgae derived BP is rapidly increasing and in this review, we highlight and discuss the current knowledge about their studies and applications in the cosmetic field.

## Bioactive Peptides in Animals and Plants

Peptides are short amino acid chains, usually ranging from 2 to 20 units, with a molecular weight under 3 kDa. As most of them possess a wide range of biological activities in living organisms, they are generally defined as bioactive peptides (BP). Due to their specific amino acid sequence and 3D conformation, BP are capable of interacting with a huge number of biological macromolecules and biochemical compounds, modulating several functions and conditions in living organisms (Moller et al., 2008). Their mode of action resembles to that of hormones and drugs, capable of activating signal transduction mechanisms in the cells, leading to an up-regulation or down-regulation of the expression of important regulatory genes. It is proven that in humans endogenous BP are involved in a wide range of therapeutic activities, such as anti-microbial, anti-hypertensive, antioxidant, anti-tumoral, anti-proliferative, hypocholesterolemic and anti-inflammatory (Sánchez and Vázquez, 2017). Besides their primary functions in triggering transduction mechanisms, they can also exert a nutritive function, providing the amino acid units as building blocks for new proteins to cells, thus contributing to the physiological protein turnover (Hartmann and Meisel, 2007).

Analogously, in plants the role of endogenous BP has been extensively studied and linked to defense response mechanisms, as well as cellular signaling during plant development stages (Schaller, 2001). A class of cationic, antimicrobial peptides (AMPs), was found to be involved in the plant innate immune response, which was one of the main mechanisms of defense against pathogens. AMPs exhibited bactericidal and fungicidal activity, thanks to their amphiphilic structure and positive charge, that facilitated the interaction and the integration into the negatively charged microbial membranes (Suarez et al., 2005). Other types of plant endogenous peptides include regulatory BP, such as systemin and enod40, whose roles in the plant wound response, mitotic activity and differentiation have been extensively reviewed (Schaller, 2001).

Signaling peptides, as plant natriuretic peptides (PNP), phytosulfokines (PSK), and rapid alkalinization factor (RAF) were purified from different plant species and it has been established their involvement in homeostatic functions (Salas et al., 2015). Another group of plant signaling peptides, members of the CLE (CLAVATA3/ESP-related) family, synthesized in the plant apical or root meristem region, were studied for their role in growth and differentiation, as well as in the maintenance of the balance between differentiation and stemness (Ito et al., 2006; Salas et al., 2015).

Nevertheless in both animals and plants many BP exist as free forms, the vast majority of known BP are produced and released following a chemical hydrolysis or enzymatic intervention, since they are encrypted in the structure of a parent protein. Thus, several classes of proteins from plant and animal origins hide active portions in their structure with specific amino acid sequences, making them potential sources of encrypted BP (Carrasco-Castilla et al., 2012; Bhat et al., 2015). The characterization of the BP released after protein hydrolysis has highlighted their wide range of biological activities related to nutraceutical and medical applications (Maestri et al., 2016). BP-rich hydrolysates, obtained from different legume plants, such as chickpea, soy bean, pea, lentil, mung bean have been studied and characterized for their antimicrobial and antihypertensive activities (Ariza-Ortega et al., 2014; Maestri et al., 2016). Either as purified forms or mixtures, BP have been proposed for the treatment and prevention of various medical conditions due to their cholesterol-lowering effects, antiprotozoal, antiviral, antithrombotic, antioxidant, antihypertensive, and antimicrobial activities (Lemes et al., 2016), and were extensively characterized as nutraceuticals (Moldes et al., 2017) and functional foods (Haque et al., 2008). BP are becoming popular in the skin and hair care field too, as they possess a wide range of biological activities in skin cells and are capable of triggering signal transduction mechanisms, leading to the activation of important gene regulators. Moreover, differently from large proteins, BP are preferred by formulators thanks to their ability to penetrate into the skin easier and to reach the deeper skin layers whether associated with efficient delivery systems (Badenhorst et al., 2014).

## Bioactive Peptides in the Cosmetic Industry

### BP From Synthetic Processes and Animal Protein Digestion

The BP that are employed in cosmetic applications come from different sources. Two of the most used methods to obtain peptides are the chemical synthesis and the partial digestion of animal proteins. Chemical synthesis involves the use of amino acid mixtures as starting material, allowing to obtain peptides with different amino acid sequence and combinations. The advantage is mostly related to the almost infinite sequences that can be intentionally assembled, giving rise to a huge range of desired functional structures.

One of the examples is the copper complexed-tripeptide GHK, which enhances the synthesis of Extra Cellular Matrix (ECM) proteins in the skin, such as collagen I, thanks to its capacity to penetrate through the stratum corneum and reach the dermis layer (Pickart and Schagen, 2015). A second example is the peptide GEKG, known as tetrapeptide-21, which was studied *in vitro* and in clinical tests for its capacity to reduce facial wrinkles (Farwick et al., 2011). The tetra-peptide increased collagen production by human fibroblast cultures and induced the expression of type I procollagen mRNA levels *in vivo*. Histochemical analysis performed on *ex vivo* skin explants confirmed that GEKG induced the production of pro-collagen, hyaluronic acid, and fibronectin. The galloyl-RGD, which is a tripeptide conjugated to gallic acid, was differently characterized for its capacity to inhibit free radical formation in HaCaT keratinocytes at the concentration of 50 ppm, and to suppress L-3,4-DihydrOxyPhenylAlanine (L-DOPA) formation and oxidation when used at higher doses (Dae et al., 2014). A Palmitoyl tetrapeptide, which represented a fragment of an immunoglobulin G, was proven to decrease IL-6 secretion in a basal setting, and served as an anti-inflammatory compound after exposure to UVB-irradiation. *In vivo* reflectance confocal microscopy studies indicated that a blend of the palmitoyl tetrapeptide and another palmitoyl oligopeptide significantly enhanced the synthesis of the ECM factors compared to placebo (Watson et al., 2009). In addition, some neurotransmitter inhibitor peptides, such as the acetyl hexapeptide three which was capable of penetrating the skin and inducing muscle relaxation, has been employed in cosmetic formulas thanks to its effect against wrinkle and fine line formation (Blanes-Mira et al., 2002).

Besides synthetic peptides, other types of peptides commonly used in skin care are those obtained by partial digestion of animal proteins, such as collagen. These peptides, generally defined with the term of matrikins (Aldag et al., 2016), have been extensively studied for their capacity to induce the production of the ECM components in the skin. KTTKS, a peptide derived from the proteolytic hydrolysis of collagen, promoted the production of fibronectin, collagen types I and III in skin fibroblasts, through the activation of TGF-β pathway (Tsai et al., 2007). A palmitoylated and more stable form of the KTTKS (pal-KTTKS) has been developed and shown to be more effective than its precursor in the collagen induction, thanks for its capacity to better penetrate the skin *stratum corneum*. The biomimetic tetrapeptide PEGP, due to the presence of the amino acids glycine and proline, was identified as a potent candidate to delay early-onset skin aging by the loss of ECM mass. Once tested, both *in vitro* and *in vivo*, it induced collagen I, elastin and fibronectin, resulting into a significant sharpening of the facial contours and wrinkle depth reduction (Maczkiewitz et al., 2018). A further example of BP derived from animal protein digestion is the mixture of peptides obtained from wool keratin. Topical formulations containing keratin-BP (MW < 1000 Da) were described as possessing moisturizing, repair-promoting and potentially radio-protective properties in the skin (Barba et al., 2008).

Instead of topical administration, a BP preparation has been studied for its benefits on the skin, once orally up-taken. The dietary supplementation of a specific bioactive collagen peptide (BCP), derived from porcine type I collagen hydrolysis, significantly reduced the wrinkles volume after 4 and 8 weeks of intake. Additionally, after 8 weeks the content of procollagen type I and elastin was higher by 65 and 18%, respectively, in the BCP-treated volunteers compared to the placebo-treated patients (Proksch et al., 2014).

### Disadvantages and Concerns of Synthetic and Animal Derived Peptides

The described examples of BP for cosmetic use, nevertheless still requested by the market, present certain drawbacks and are not fully accepted by all the consumers, mostly due to the risks associated with the presence of contaminating chemicals used in the process (for chemical synthesis) or the use of animal derived substances (for digested products). The use of chemical reactions to synthesize peptides implies the use of toxic solvents and reagents in the cycle and, although the last step of the process involves the elimination of most chemicals, the final product can still carry contaminating solvent residues or impurities (Guzman et al., 2007; D’Hondt et al., 2014). Even the solid-phase peptide synthesis, the most used manufacturing procedure for drug peptides today, can generate adducts, impurities and unwanted peptide counter ions, such as trifluoroacetate, which can be hardly eliminated from the final peptide product (D’Hondt et al., 2014).

On the other hand, the use of animal proteins in enzymatic digestions implies very strict veterinary controls in the animal source in order to exclude infections from any kind of pathogenic virus, or even the presence of dysfunctional protein aggregates, that can cause very serious diseases in human as well, such as the case of the Bovine Spongiform Encephalopathy (BSE) in cattle. Nevertheless of the several check points during the whole production process, certain risks associated with the presence of animal derived harmful substances cannot be completely abolished (Yokoyama and Mohri, 2008). Besides the safety concern, which certainly represents the most relevant priority for cosmetic brands and consumers, the whole tendency of the market is going toward eco-certified plant derived ingredients. Plant-extracted compounds are becoming always more requested by the market, both because of the trends associated with the idea of well tolerated and less allergenic ingredients, and because strict vegan consumers are progressively increasing among the global world population. For Muslims, for example, additional rules for Cosmetics imply further considerations relative to the religion, which restrict or prohibit any contact with products that are inconsistent with Islam laws. In relation, Halal certification is required for many cosmetic ingredients to attest the product conformity during its whole production cycle with the special attention to the animal origin and the processing (Rigano, 2017). Both the concerns and the preferences toward plant derived material has prompted skin care ingredient producers to substantially invest on alternative ways to obtain BP, which can be analogously effective in terms of biological activity as their animal-derived or synthetic counterparts, and fully guaranteed for safety and sustainability.

### Plant and Microalgae-Derived Peptides for Cosmetic Applications

All plant and microalgae-derived peptides used in Cosmetics today, in particular in the skin care market, are formulated as mixtures of many different protein fragments, obtained from hydrolysis of bigger proteins, using either the chemical or enzymatic methods (Table 1). By choosing a specific protein source, and by modulating the extent and the conditions of the hydrolytic process, it was possible to obtain BP of different nature, length and composition, adapted to fulfill desired biological functions. All the examples that we have reported and discussed hereafter refer to raw peptide preparations, often containing other plant cell metabolites, whose activity has been characterized, either *in vitro* or *in vivo*, for their capacity to activate significant biological functions in skin cells, acquiring promising potentialities to be developed as cosmetic active ingredients.

**TABLE 1:** Examples of plant and microalgae derived peptides used in cosmetics.

**Source**	**Type of bioactive peptide (BP) preparation**	**Main activities**	**References**
Plants			
*Avena sativa*	Enzymatic hydrolysates	Reduced the oxidative stress-induced cell injury in skin fibroblasts	Feng et al.,2013
*Glycine max*	Total lysates	Improved wound healing by increasing ECM synthesis in the skin, promoting cell adhesion	Chien et al.,2013
*Triticum vulgare*	Chemical/enzymatic hydrolysates	Improved skin healing capacity; anti-inflammation activity by reducing NO, IL-6, TNF-alpha; PGE-2	Burnett et al.,2018
*Solanum tuberosum*	Preparations obtained by yeast fermentation	Stimulated lipid metabolism in human aged keratinocytes	Popa et al.,2006
*Nicotiana sylvestris* cell cultures	Chemical/enzymatic hydrolysates of plant cell wall glycoproteins	Inhibited oxidative damages in skin cells, induced DNA repair factors (GADD45alfa) and longevity markers (Sirtuins)	Apone et al.,2010
*Brassica rapa* hairy root cultures	Chemical/enzymatic hydrolysates of plant cell wall glycoproteins	Depigmentation activity by down regulation of MITF expression in epidermis cells	Sena et al.,2017
*Lotus japonicus* cell cultures	Chemical/enzymatic hydrolysates of plant cell wall glycoproteins	Anti-aging activity by inducing GDF11 production and consequent collagen and periostin restoration in skin fibroblasts	Tito et al.,2019
Microalgae			
*Chlorella pyrenoidosa*	Isolated peptide fractions (430–1350 Da)	Inhibited UVB-induced MMP-1 expression in skin fibroblasts by suppressing AP-1, CYR61 and MCP production	Chen et al.,2011
		Reduced UVC-induced cytotoxicity by modulating Caspase-3, FADD and ADP-ribose-PARP-1	Shih and Cherng,2012
*Arthrospira platensis* (Spirulina)	Enzymatic hydrolysates	Promoted hydration in skin cells by increasing AQP3, HAS3, and Filaggrin expression; counteracted osmotic stress damage by modulating Smith gene expression	De Lucia et al.,2018

An *Avena sativa* (oat) peptide-rich preparation, obtained from oat bran by enzymatic hydrolysis, was tested *in vitro* on H_2_O_2_-stressed dermal human fibroblasts and the results demonstrated that the preparation was effective to reduce oxidative stress-induced cell injury, through an enhanced activity of the enzymes SODs and an inhibition of the malondialdehyde (MDA) levels. Although further exploration about the skin care potential *in vivo* might be necessary, the oat BP preparation was proposed as functional ingredient in anti-aging skin creams to help preventing damages induced by oxidative stress and UV (Feng et al., 2013).

In recent studies, the wound healing potentialities of a *Glycine max* (soy) lysate, containing BP with amino acid sequences similar to those of ECM proteins present in the human skin, was explored (Tokudome et al., 2012). These ECM-mimetic peptides produced in soy improved wound healing by increasing dermal ECM synthesis, stimulating re-epithelialization, promoting cell adhesion and supporting tissue regeneration (Chien et al., 2013). With the main goal of increasing the number of applicative outputs, other authors developed a plant-based nanofibrous cellulose acetate scaffold, supporting a layer of soy BP extract (Ahn et al., 2018). The scaffold membranes, combined with the soy BP hydrolysate, successfully mimicked the physiological properties of the native skin and promoted fibroblast proliferation, migration and infiltration *in vitro*. *In vivo*, the scaffolds accelerated re-epithelialization and epidermal thickening, as well as reduced scar formation and collagen mis-assembly during the wound repair stages. These findings provided a valuable example of how plant-derived BP could be employed in a next generation of regenerative dressings to push the envelope of nanofiber technology in the skin care market.

Protein hydrolysates from *Triticum vulgare* (wheat) have also been produced and proposed as skin and hair-conditioning ingredients in personal care products (Burnett et al., 2018). The BP contained in the hydrolysate, obtained by acidic, enzymatic or other chemical digestion procedures, have shown to be very effective *in vitro* in accelerating skin healing and in reducing the release of several inflammatory mediators, such as nitric oxide (NO), Interleukin 6 (IL-6), Tumor Necrosis Factor (TNF) -alpha, and ProstaGlandin E2 (PGE-2) (Sanguigno et al., 2018). To prevent skin sensitization reaction in allergic subjects due to wheat gluten-derived peptides (Adachi et al., 2012), other authors showed that, when the hydrolysis was carried out at longer times, the wheat BP did not exceed 30 amino acid in length and, in contrast to those with average MWs > 10 KDa, they did not elicit any hypersensitivity reactions in sensitized individuals, still keeping their biological activity (Burnett et al., 2018).

A *Solanum tuberosum* (potato) protein hydro-lysate provided an additional example of how BP, generated by a hydrolytic process, activated response mechanisms in skin cells and could be advantageously applied to skin care Cosmetics. The potato hydrolysate stimulated the lipid metabolism in skin keratinocytes when added to the culture medium (Popa et al., 2006), and induced a stronger increase of the long-chain fatty acid-containing glucosylceramides in aged keratinocytes than in younger ones (Popa et al., 2010). Moreover, besides the effect on glucosylceramide synthases, it was found that the amount of the neutral phospholipids and the gangliosides was significantly induced by the BP-containing potato hydrolysate, suggesting an activation of the PPARs (peroxisome proliferation activated receptors), that are known to trigger the expression of many lipid biosynthesis genes in keratinocytes (Man et al., 2006).

Similar to plants, plant tissues grown as suspension cultures and obtained through green biotechnological approaches represent an even more valuable source of BP. Besides being free of pathogens, pollutants and agrochemical residues, which could contaminate most of the plant-derived extracts, plant tissue cultures rarely contain toxic compounds or potential allergens (Apone et al., 2017). Moreover, plant cells, grown as suspension cultures, are particularly rich in cell wall proteins, whose production is enhanced during the culture formation and the developmental process that occurs when pieces of plant tissue are put in liquid media to form cultures (Apone et al., 2007, 2017). The most abundant proteins present in the cell wall are those belonging to the main classes of hydroxy-proline-rich glycoproteins (HRGPs), proline-rich proteins (PRPs), glycine-rich proteins (GRPs), and arabinogalactan proteins (AGPs), with high content of glycine and proline (Ringli et al., 2001), which are also the most abundant amino acids in human collagen. A mixture of BP, together with sugars derived from the hydrolysis of the glycoprotein saccharides, was obtained from the digestion of *Nicotiana sylvestris* cell wall glycoproteins and extensively investigated *in vitro* for its activity to inhibit oxidative damages in skin cells, as well as for the capacity to induce DNA repair factors, such as the Growth Arrest and DNA-Damage-inducible protein 45 alpha (GADD45α) and the longevity markers, Sirtuins (Apone et al., 2010). In addition, the cell wall derived BP stimulated collagen I and collagen III synthesis in human fibroblasts, and significantly inhibited the metallo-proteinases 1, 3, and 9 production, suggesting promising skin anti-aging properties for the cosmetic market. Even though the role of the sugars in skin care has not been fully clarified, several evidences indicated that the sugar fraction contained in cosmetic preparations had beneficial effects on hydration and had an anti-inflammatory effect on dermal cells (Tolg et al., 2014).

An analogous preparation obtained from *Brassica rapa* root cultures was studied for its capacity to reduce the accumulation of melanin in skin cells both *in vitro* and *ex vivo*, by inhibiting the activity and the expression of the enzyme tyrosinase, main responsible of the melanin synthesis in melanocytes (Sena et al., 2015, 2017). The effect of the BP was that of negatively affecting the melanogenesis signal transduction mechanism, by down-regulating MITF expression, a key transcription factor involved in the melanin pathway. The depigmenting activity produced by the preparation was also evaluated on skin explants, confirming a significant reduction of melanin content both in the sub-basal and in the basal epidermis layers (Sena et al., 2017).

In a more recent study a peptide/sugar preparation, obtained from the cell walls of *Lotus japonicus* cell cultures, was characterized for the presence of proline, hydroxyproline and leucine as the main amino acid components and was proposed as anti-aging ingredient for the skin care, thanks to its capacity to induce the expression and the production of a the cell rejuvenating growth factor GDF11 *in vitro* and a consequent restoration of some ECM component production level, such as collagens and periostin (Tito et al., 2016, 2019).

The chemistry and biological activities of BP obtained from marine algae have been investigated, although they have not been as well characterized as the peptides from other sources (Fan et al., 2014). A number of BP have also been obtained by hydrolysis of microalgae proteins but few of them have been proposed for skin care applications. Several microalgae species have got high protein content, up to 47% of the dry weight, compared to the terrestrial plants, and many of them evolved unique protein sequences and structures thanks to their adaptations to more complex habitats, such as those related to extreme conditions (Bimonte et al., 2016; Berthon et al., 2017).

Among the large variety of microalgae species, the genus *Chlorella* and *Arthrospira* (commonly known as Spirulina) certainly represent the most established and well known sources of compounds in the skin care market. The freshwater grown unicellular alga *Chlorella* was already known for the different biological effects on human cells, such as boosting immune functions (Suarez et al., 2010), accelerating dioxin elimination (Morita et al., 1999) and preventing the stress-induced ulcers (Tanaka et al., 1997). Recently it was established that the *Chlorella* derived peptide (CDP) fraction, with molecular distribution of 430–1350 Da, diminished UVB-induced matrix metalloproteinase-1 (MMP-1) and cysteine-rich 61 (CYR61) gene expression and monocyte chemoattractant protein-1 (MCP-1) production in UV-B irradiated fibroblasts (Chen et al., 2011). Moreover, the authors reported that the UVB-suppressed pro-collagen and TbRII (Transforming growth factor, TGF-β, receptor) mRNAs were restored by CDP treatment. Moreover, CDP inhibited UVB-induced MMP-1 expression in skin fibroblasts by reducing the expression of the activator protein-1 (AP-1), CYR61 and MCP-1 (Chen et al., 2011). Further studies by the same authors revealed also the protective effects of CDP against UVC-induced cytotoxicity through the inhibition of the caspase-3 activity, and reduced the expression of the phosphorylated Fas associated death domain (FADD) and the poly (ADP-ribose) polymerase-1 (PARP-1) (Shih and Cherng, 2012).

Analogously, the microalga *Arthrospira platensis* (Spirulina), belonging to the group of blue-green Cyanobacteria, is a consistent source of protein, being approximately 60% of its entire weight (Shabana et al., 2017). A BP-enriched fraction obtained from Spirulina was originally studied for its pharmacological activity on the angiotensin I-converting enzyme (ACE) and its anti-proliferative effect in lung cancer cells (Lu et al., 2010; Czerwonkaa et al., 2018). More recently, it was studied the skin care related activity of two different protein hydrolysates from Spirulina, an extract obtained by microbial fermentation (SEF) and extract obtained by enzymatic digestion (SED), and compared to a total undigested extract (SE) (De Lucia et al., 2018). SDS–PAGE analysis of the extracts was performed and the C-phycocyanin was used as indicator of the degree of protein hydrolysis and generation of BP. It was demonstrated that the SED extract, containing protein derived BP, was the most effective in promoting skin hydration and in fighting skin osmotic stress damages *in vitro*, by increasing the gene expression of factors specifically involved in the water balance maintenance in keratinocytes, such as the aquaporin3, hyaluronic acid synthase 3 and filaggrin. Moreover, the extract was able to abolish ROS induction caused by oxidative stress agents, and to counteract osmotic stress damage by modulating Smit gene (Na+/myo-inositol cotransporter), that was responsible for an increased uptake of osmolytes (De Lucia et al., 2018).

### Limitations in the Use of Plant and Microalgae-Derived Peptides

As reported in Table 1, the most used plant and microalgae peptides in Cosmetics are obtained by protein hydrolysis and consist of mixtures of peptides of different sizes. Although these hydrolysates may have a broad range of biological activities thanks to their complex nature, their use may be limited in Cosmetics whether a more specific biological function is preferred. The choice of a specific peptide with a known amino acid sequence and length can be sometimes suitable when the interaction with a single and defined target is desired, thus avoiding interactions with other unspecific biological components. This is particularly true in the Pharmaceutical field, where the employment of peptides as drugs is in perfect agreement with the aim of modulating a target-molecule without disturbing other biochemical functions and avoiding undesired side-effects. Analogously, the study of a single peptide activity could be preferred in some cosmetic applications as well, but the isolation of a single peptide fraction from plant and microalgae hydrolysates may become very challenging. Moreover, the most frequently used technologies to isolate peptides from more complex mixtures still include chromatography and pressure-driven filtration processes, which would increase the final cost of the products, making them no longer sustainable for the cosmetic industry (Fan et al., 2014).

An additional limitation could be associated with the risk of potential allergens, since most of the peptide preparations are produced as unpurified mixtures of several components (Jack et al., 2013). Plant and microalgae hydrolysates may contain allergens and potentially toxic contaminating compounds, whose presence must be necessarily certified. Thus, to ensure conformity to the quality standards and answer the safety concerns, the hydrolysates must be subjected to chemical analysis once they are developed as new active ingredients for cosmetic applications. In other case, additional studies on how to prevent and limit the possible allergic reactions due to identified chemicals may even become necessary (Goossens, 2011). For these reasons, in Cosmetics peptide preparations deriving from plant tissue cultures have today become preferable to common plant extracts, because they derive from biological sources grown in the laboratory, under controlled and axenic conditions, where the risk of the presence of potential allergens and environmental pollutants is almost completely abolished.

## Conclusion and Future Remarks

As described through the reported examples, BP, deriving from plant and microalgae sources, possess a broad spectrum of biological activities and their uses as ingredients in skin care and cosmetic products are acquiring more and more opportunities.

Moreover, once derived from plants or microalgae, BP can be used as combinations and mixture with other metabolites in cosmetic formulas, and can exert their biological effects with very low risk of inducing allergenic reaction or undesired side effects.

Despite of the significant progress in the isolation and the characterization of BP from natural sources, as well as the assessment of their biological activities, there are still some aspects, as their production at large scale and the activity maintenance in formulas, which would need further studies in order to extend the range of applications and fulfill the requirements of the continuously growing cosmetic market.

As future development, the plant and microalgae can provide even more innovative opportunities to develop new products for the cosmetic market, thanks to their enormous versatility: for example, new species may represent a valuable source of unexplored BP with novel chemical features and unexpected biological properties. In addition, many plant and microalgae species can be employed as natural bio-factories of recombinant protein and peptides with specific sequences and structures, whose production by chemical synthesis may present some limitations. Indeed, thanks to the recent progresses of Molecular Biology, the expression of proteins and peptides in plant and microalgae by using genetic transformation has become a common practice. Although the employment of genetically modified organisms to produce recombinant compounds has been adopted in Medicine for years, in Cosmetics the feedback of the consumers about this topic has not been totally positive so far, probably because of their limited understanding and unfamiliarity about this issue, and certainly the unclearness of the current legislation is not helping either. Nevertheless, proteins and BP produced in plant and microalgae systems can be considered even safer than those produced through conventional methods, thus they have got all the right credentials to be adopted as ingredients in different human health care products.

## Author Contributions

All authors conceived and designed the manuscript, and revised, read, and approved the final version of the manuscript.

## Conflict of Interest Statement

AB was employed by the company Arterra Bioscience srl. The remaining authors declare that the research was conducted in the absence of any commercial or financial relationships that could be construed as a potential conflict of interest.
